# On the role of ethylene, auxin and a GOLVEN-like peptide hormone in the regulation of peach ripening

**DOI:** 10.1186/s12870-016-0730-7

**Published:** 2016-02-11

**Authors:** Alice Tadiello, Vanina Ziosi, Alfredo Simone Negri, Massimo Noferini, Giovanni Fiori, Nicola Busatto, Luca Espen, Guglielmo Costa, Livio Trainotti

**Affiliations:** Dipartimento di Biologia, Università di Padova, Viale G. Colombo 3, I-35121 Padova, Italy; Dipartimento di Colture Arboree, Università di Bologna, Viale Fanin 46, 40127 Bologna, Italy; Dipartimento di Scienze agrarie ambientali – Produzione – Territorio – Agroenergia (Di.S.A.A), Università degli Studi di Milano, via Celoria 2, Milan, I-20133 Italy; Present addresses: Research and Innovation Centre, Fondazione Edmund Mach, Via Mach 1, 38010, San Michele all’Adige Trento, Italy; Present addresses: BIOLCHIM S.p.A., Via San Carlo 2130, 40059 Medicina, BO Italy; Present addresses: FA.MO.S.A s.r.l., Via Selice 84/A, 40026 Imola, BO Italy; Present addresses: Dipartimento di Colture Arboree, Università di Bologna, Viale Fanin 46, 40127 Bologna, Italy

**Keywords:** 1-methylcyclopropene (1-MCP), Index of absorbance difference (I_AD_), Microarray, Nectarine, *Prunus persica*, Hormone peptide, GOLVEN, ROOT GROWTH FACTOR

## Abstract

**Background:**

In melting flesh peaches, auxin is necessary for system-2 ethylene synthesis and a cross-talk between ethylene and auxin occurs during the ripening process. To elucidate this interaction at the transition from maturation to ripening and the accompanying switch from system-1 to system-2 ethylene biosynthesis, fruits of melting flesh and stony hard genotypes, the latter unable to produce system-2 ethylene because of insufficient amount of auxin at ripening, were treated with auxin, ethylene and with 1-methylcyclopropene (1-MCP), known to block ethylene receptors. The effects of the treatments on the different genotypes were monitored by hormone quantifications and transcription profiling.

**Results:**

In melting flesh fruit, 1-MCP responses differed according to the ripening stage. Unexpectedly, 1-MCP induced genes also up-regulated by ripening, ethylene and auxin, as CTG134, similar to GOLVEN (GLV) peptides, and repressed genes also down-regulated by ripening, ethylene and auxin, as CTG85, a calcineurin B-like protein.

The nature and transcriptional response of CTG134 led to discover a rise in free auxin in 1-MCP treated fruit. This increase was supported by the induced transcription of CTG475, an IAA-amino acid hydrolase. A melting flesh and a stony hard genotype, differing for their ability to synthetize auxin and ethylene amounts at ripening, were used to study the fine temporal regulation and auxin responsiveness of genes involved in the process. Transcriptional waves showed a tight interdependence between auxin and ethylene actions with the former possibly enhanced by the GLV CTG134. The expression of genes involved in the regulation of ripening, among which are several transcription factors, was similar in the two genotypes or could be rescued by auxin application in the stony hard. Only GLV CTG134 expression could not be rescued by exogenous auxin.

**Conclusions:**

1-MCP treatment of peach fruit is ineffective in delaying ripening because it stimulates an increase in free auxin. As a consequence, a burst in ethylene production speeding up ripening occurs. Based on a network of gene transcriptional regulations, a model in which appropriate level of CTG134 peptide hormone might be necessary to allow the correct balance between auxin and ethylene for peach ripening to occur is proposed.

**Electronic supplementary material:**

The online version of this article (doi:10.1186/s12870-016-0730-7) contains supplementary material, which is available to authorized users.

## Background

The transition from maturation to ripening in fleshy fruits can be either dependent on the hormone ethylene or not. In the first case fruit, such as peaches, tomatoes, bananas and apples exhibit a characteristic respiratory rise and are defined climacteric, in the second case do not and are classified as non-climacteric (e.g. strawberry, grape, citrus). It is known that climacteric fruit can produce ethylene by either a system-1 or a system-2 biosynthesis, with the latter active when autocatalytic ethylene is produced [1, 2]. System-2 ethylene has been shown to modulate the expression of hundreds of genes both in tomato [3] and in peach [4]. All plant tissues are able to produce ethylene and the gaseous hormone is involved in many developmental processes [5] and in response to both biotic [6] and abiotic stresses [7, 8]. In the model plant Arabidopsis there are nine 1-aminocyclopropane-1-carboxylic acid (ACC) synthase (ACS, [9]) and five ACC oxidase (ACO, http://www.arabidopsis.org) genes, coding for different isoforms of the two enzymes involved in the conversion of S-adenosyl-methionine (AdoMet) to ethylene. The unique and overlapping roles of the different members of the Arabidopsis ACS family have been investigated both at molecular [9] and biochemical [10] levels.

In the tomato genome, the model plant for fleshy fruit ripening, eleven ACS and seven ACO putative genes were identified, of which *LeACS1A*, *LeACS2*, *LeACS4*, *LeACS6*, *LeACO1*, *LeACO3* and *LeACO4* are differentially expressed during ripening (reviewed in [11, 12]). A possible auxin promoting effect on system-2 ethylene production in tomato fruit has not been considered in the model explaining the transition from system-1 to system-2 ethylene biosynthesis [13], even though the inductive effect of auxin on ACS transcription in vegetative tissues has long since been known [14].

The induction of *LeACS4* by auxin, even in tomato plants with down-regulated expression of the *DR12* gene, coding for an Auxin Responsive Factor (ARF), has been shown to occur also in maturing fruit [15]. Nevertheless, auxin induction of ethylene synthesis in ripening fruit did not draw much attention, presumably because auxin has normally been considered to counteract ripening (see, for example, [16]). In peach a transcriptomic approach has highlighted a previously underestimated role of auxin in the regulation of fruit ripening [4]. The requirement of auxin to switch to system-2 ethylene production in fruit was later shown to be the reason of the stony-hard phenotype, as fruit from this genotype was found to be unable of rising IAA concentration [17]. However, being the auxin-ethylene relationship very intricate, several overlapping effects are still to be assigned to either one or the other of the two hormones.

The synthetic compound 1-methylcyclopropene (1-MCP) is structurally related to ethylene and widely used on many species to block its unwanted effects, as in fruit ripening and in cut flowers [18]. It has been shown that 1-MCP interacts with both ETR1 and ERS1 proteins, thus stabilizing their repressor activity [19], and for such a reason this chemical is commercially used to delay hormone’s unwanted effects. As system-2 ethylene synthesis is autocatalytic, 1-MCP should block it, and this is what has been reported in many fruit, such as apple, tomato and banana (reviewed in [18]). In peach there are contrasting reports: some researchers state that 1-MCP can block ethylene synthesis, and thus delay fruit ripening [20, 21], although not efficiently [22], while others found enhanced ethylene production [23–25].

By using a non destructive spectroscopic index (index of absorbance difference, I_AD_) which can be used to asses the exact maturation and ripening phase of peach fruits [26] also in stony-hard genotypes [27], we could perform 1-MCP and auxin treatments on homogeneously ripe fruits. The possibility of sorting fruits in a precise series of ripening stages has made it possible to gain new findings on the regulation of this transition by auxin and ethylene and on 1-MCP action in peach. More interestingly, this experimental system resulted to be suitable to shed new light on the regulation of ethylene synthesis and its cross-talk with auxin, possibly mediated and/or enhanced by a peptide hormone belonging to the RGF/GLV (ROOT GROWTH FACTOR/GOLVEN) family.

## Results

### Effect of 1-MCP on fruit ripening

In order to perform 1-MCP treatments on fruit at a homogeneous stage of ripening, the index of absorbance difference, (I_AD_, [26]) was used to group melting flesh peaches according to their maturity and ripening stage.

**Fig. 1:**
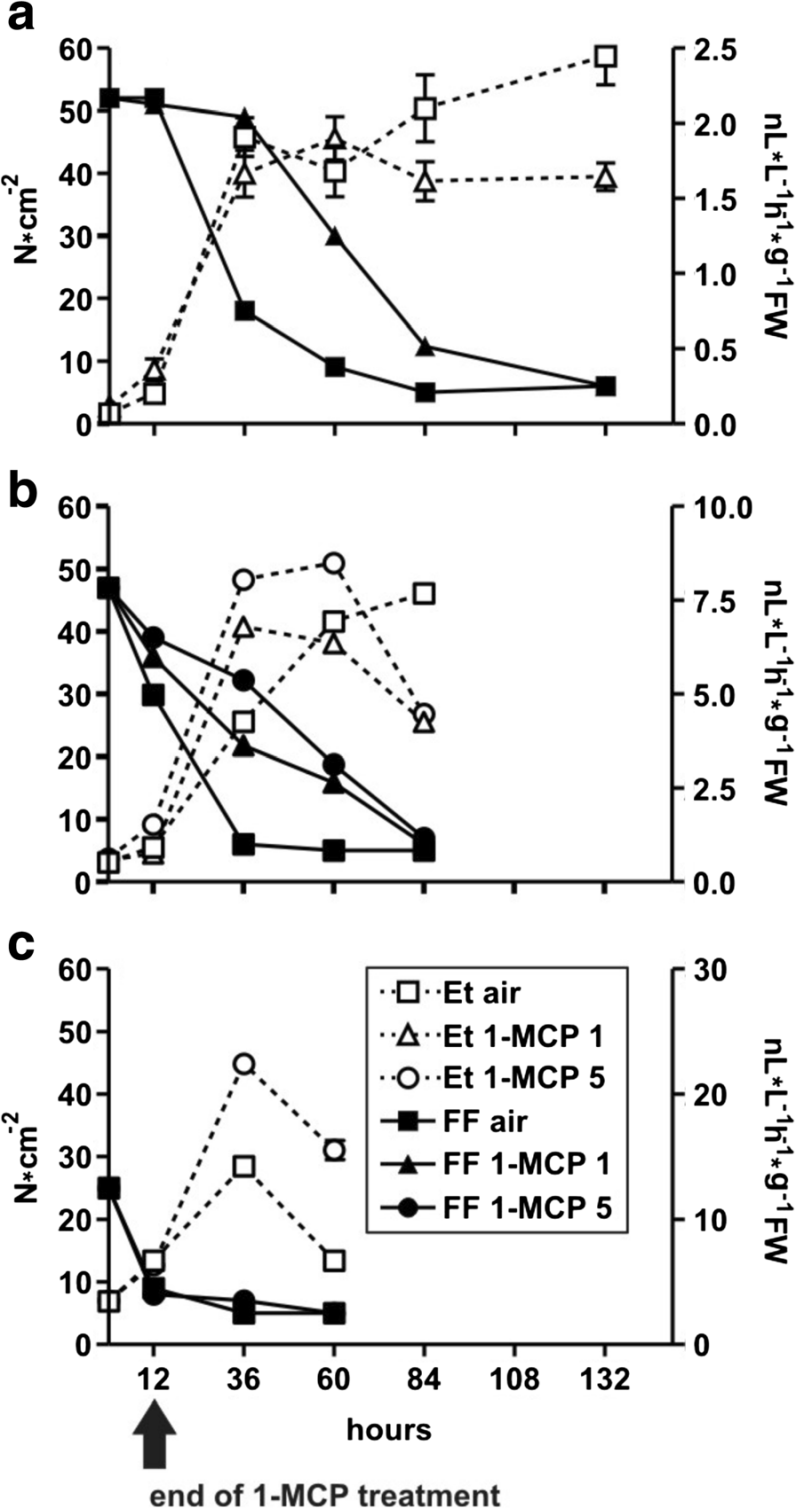
Flesh firmness (solid lines, filled symbols, left Y axe) and ethylene production (dashed lines, open symbols, right Y axe) during post-harvest of peaches either treated (1-MCP) or not (air) with 1-MCP (1 or 5 μL L^−1^). The Y scale is the same in the three panels for FF (left), while it differs for ethylene production (right). I_AD_ was used to group S4 fruit according to their ripening stages: class 0 (pre-climacteric, panel (**a**)), class 1 (onset of climacteric, panel (**b**)), and class 2 (climacteric, panel (**c**)). The arrow at the bottom indicates the end of the 1-MCP treatment in 1-MCP-exposed fruit. Thereafter, fruit were kept in air at 25 °C. Data represent the mean (*n* = 40) ± S.D

The efficacy of 1-MCP in delaying peach ripening was determined by evaluating ethylene production and flesh firmness (FF, Fig. 1). As fruits belonging to class 1 and 2 were already producing ethylene, treatments were performed with both 1 and 5 μL L^−1^ of 1-MCP (class 1) or with 5 μL (class 2), to saturate all possible hormone binding sites. 1-MCP effect was different depending on the class. In class 0 1-MCP was effective in both reducing ethylene production (Fig. 1a, broken lines) and delaying softening (Fig. 1a, solid lines). In class 1 1-MCP effect was intermediate; indeed, the inhibitor speeded up ethylene production (Fig. 1b, broken lines) but was able to delay fruit softening (Fig. 1b, solid lines). The experiment was stopped after 84 h because of fruit decay. In class 2 1-MCP induced ethylene production (Fig. 1c, broken lines) and was ineffective on fruit softening (Fig. 1c, broken lines). The experiment was stopped after 60 h because of fruit decay.

### Effect of 1-MCP on gene transcription

The effects of 1-MCP on the peach fruit transcriptome were evaluated by a microarray approach using the μPEACH1.0 platform [28]. Class 0 fruit kept in air for 24 h after the 1-MCP treatment (i.e. 36 h after harvest) were used because they showed the highest retention of FF compared to control fruit. A direct comparison approach (i.e. “36 h air” vs “36 h 1-MCP”) was employed.

Setting the False Discovery Rate (FDR) to 5 %, 121 probes resulted to be differentially expressed (58 down-regulated, 63 up-regulated; see Additional file 1 for the complete list). These data are partially overlapping to those obtained with the same μPEACH1.0 platform [25].

### 1-MCP effect on genes regulated by ripening and ethylene

Microarray data were crossed with those already available on the regulation of peach ripening and exogenous ethylene application [4]; this analysis highlighted:

i) 20 probes induced by both ripening and ethylene and, as expected, repressed by 1-MCP. These included genes encoding an endopolygalacturonase (PG, CTG420), a pyruvate decarboxylase (PD, CTG112) and a nine-cis-epoxycarotenoid dioxygenase (NCED, CTG2980), whose expression profiles was confirmed by quantitative reverse transcriptase real-time PCR (qRT-PCR, see Additional file 2, A, B and C).
ii) 18 probes that were down-regulated by both ripening and ethylene but up-regulated by the 1-MCP treatment. Among them were genes encoding a plasma membrane intrinsic protein (PIP, CTG349), a sorbitol transporter (ST, CTG2902) and a RD22-like protein (CTG974), whose expression profile was confirmed by qRT-PCR (, see Additional file 2, D, E and F).

Noteworthy is that there were not genes induced by ripening, ethylene and 1-MCP nor repressed by the same conditions.

### 1-MCP effect on genes regulated by ripening and auxin

As done for ethylene, microarray data were crossed with those already available on the regulation of peach ripening by auxin [4]; this analysis highlighted:

i) 11 probes induced by both ripening and auxin and repressed by 1-MCP. All these 11 probes fell within the group of those 20 induced by ripening and ethylene and repressed by 1-MCP seen above, thus confirming that their auxin responsiveness was mediated by ethylene.
ii) 13 probes behaved in the opposite way, that is, they were down-regulated by both ripening and auxin but up-regulated by the 1-MCP treatment. Of these, 11 were in common with the 18 probes down-regulated by ripening and ethylene and up-regulated by 1-MCP, thus confirming that also for these genes their auxin responsiveness was mediated by ethylene.

**Fig. 2:**
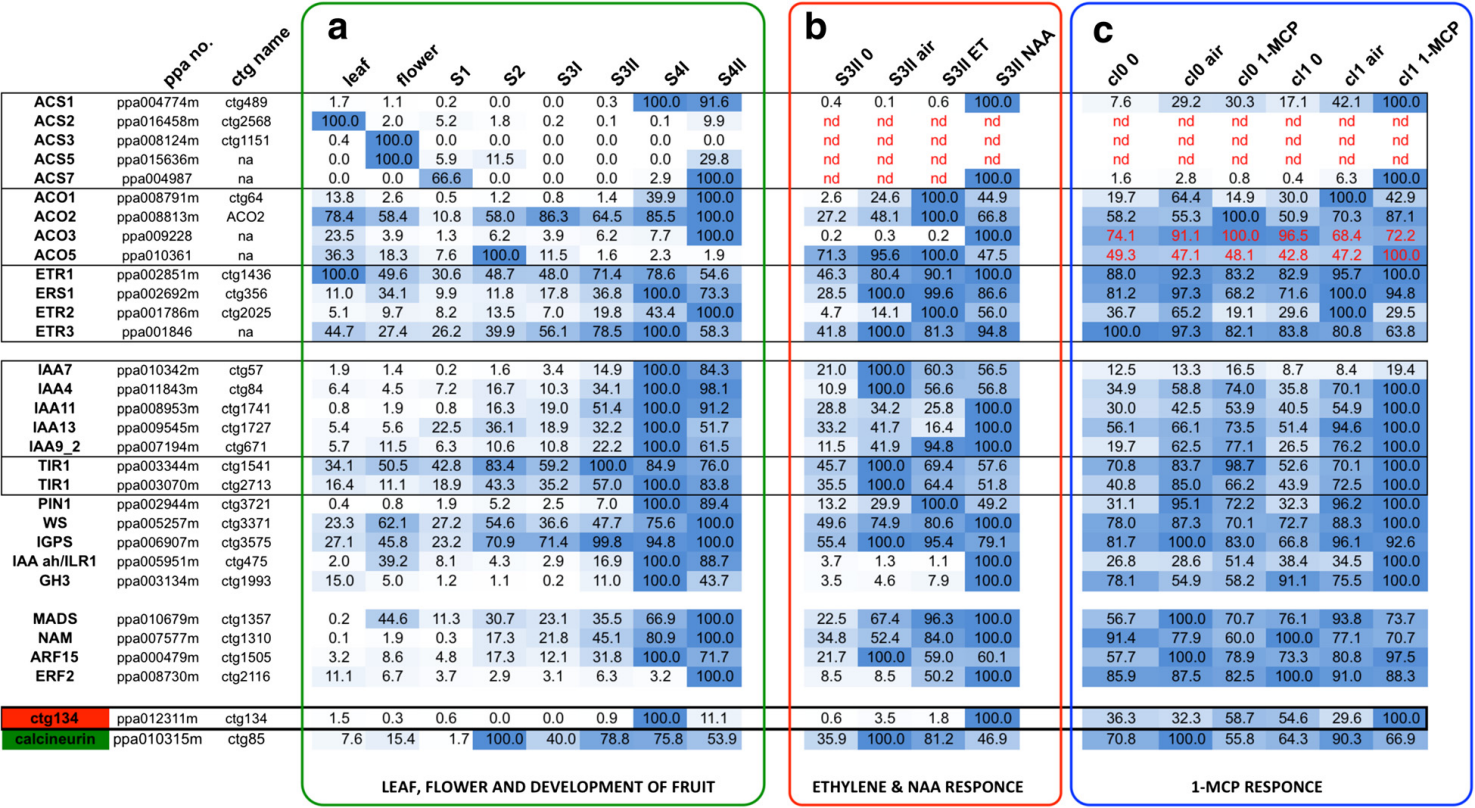
Relative expression profiles of selected genes in leaf, flower and fruit at different stages of development (S1, S2, S3I, S3II, S4I, and S4II, corresponding to 40, 65, 85, 95, 115 and 120 days after full bloom, respectively; sector A), in fruit at S3II following ethylene (ET) and NAA treatment (sector B) and in preclimacteric S4 fruits belonging to class 0 (cl0) or class 1 (cl1) treated with 1-MCP (sector C). Genes belonging to the ethylene domain (upper group), auxin domain (second group), transcription factors (third group) or with the unexpected transcriptional response following 1-MCP treatment are grouped. Genes belonging to the same family are boxed. Expression values, determined by qRT-PCR, were related to the highest expression of each gene (100 %, blue) within each experiment (**a**, **b** carried out with RH samples and **c**, carried out with SRG fruits; both RH and SRG produce melting flesh fruits). ppa no. indicate the peach gene identifier as described in [30], while CTG name indicate the cDNA identifiers on the microarray μPEACH1.0 as described in [28]. Hormone treatments (ET: ethylene; NAA: 1-naphthalene acetic acid, a synthetic auxin) lasted for 48 h (group B). SRG fruits were collected at commercial maturity date and sampled after 36 h of storage either in air or in 1-MCP (12 h) plus air (i.e. 24 h in air after the end of the 1-MCP treatment; group C)

Noteworthy is that microarray analysis highlighted only one gene as induced by ripening, auxin and 1-MCP (CTG134, encoding a predicted hormone peptide) and also only one gene as repressed in the three situations (CTG85, encoding a calcineurin B-like protein). This unexpected expression profile was confirmed by qRT-PCR for both CTG134 and CTG85 (Fig. 2).

### Regulation of system-2 ethylene biosynthesis

The increase in system-2 ethylene production measured in 1-MCP treated fruit of class 1 and 2 led us to investigate the regulation of hormone metabolism during the transition from developing to ripening fruits. To better understand the function of the considered genes, their expression was evaluated, by means of qRT-PCR experiments, in fruits at different developmental stages and in non-fruit tissues such as leaf and flower; furthermore, their responsiveness to exogenous ethylene and 1-naphthalene acetic acid (NAA, an auxin analogue) was evaluated at the pre-climacteric stage (S3II treated fruit;[4]).

### Transcriptional regulation of ethylene biosynthetic genes

Beside the three known ACS genes [20, 29], probes for five additional members of this family were designed based on EST searches and the recently released peach genome sequence [30]. A comparison with Arabidopsis *ACS* genes allowed us to assign *ACS1* (CTG489, ppa004774m) and *ACS2* (CTG2568, ppa016458m) to group A [9], and *ACS3* (ppa008124m), *ACS5* (ppa015636m), *ACS7* (ppa004987m) and *ACS8* (ppa022214m) to group B. Furthermore, *ACS4* (CTG5158, ppa003908m) and *ACS6* (ppa004475) clustered with Arabidopsis *AtACS10* and *AtACS12* (Additional file 3) and thus most likely are aminotransferases that do not act on branched chain amino acids and do not have ACC synthase activity [31]. Therefore, they were not considered further. The expression of *ACS8*, if any, was below the detection limit in the tested samples.

As previously described [4], *ACS1* (CTG489) transcription was dramatically induced by ripening (i.e. the passage from S3II to S4I, Fig. 2a). In pre-climacteric S3II peaches NAA was much more effective than ethylene in increasing ACS1 mRNA abundance (Fig. 2b). Blocking ethylene perception with 1-MCP seemed ineffective on ACS1 accumulation in class 0 fruits, while *ACS1* was strongly induced in class 1 fruits (Fig. 2c).

*ACS2* (CTG2568) expression was relatively abundant only in fully developed leaves, but it was very low in fruit, with a peak at the beginning of development (S1, reported also in [17] and a maximum in senescence (i.e. S4II, Fig. 2a). ACS2 mRNA was almost undetectable in S3II and S4I fruits, thus ethylene, NAA and 1-MCP responsiveness could not be assessed (Fig. 2b and c).

ACS3 mRNA (CTG1151) was detected only in flowers and leaves (Fig. 2a), and, although peaking in the former, it was only a fraction of ACS1 and ACS2 expression (not shown, from absolute quantification data used to build Fig. 5).

*ACS5* was expressed at extremely low levels (comparable to those of *ACS3*) in flowers and very young fruits (S1 and S2; Fig. 2a). In ripening fruits its expression was hardly detectable, also after treatments with ethylene, NAA and 1-MCP (data not shown).

*ACS7* expression was also very low and detectable only in S1 and S4 fruit, with a maximum in S4II (Fig. 2a). NAA had a positive effect on ACS7 mRNA accumulation (Fig. 2b) as 1-MCP had on class 1 fruit (Fig. 2c).

As regards the ACC oxidases (ACOs), the well-known ripening and ethylene induced expression of *ACO1* (CTG64, [32]) as well as its repression by 1-MCP [25] was confirmed (Fig. 2). *ACO1* transcription’s dependency on ethylene was strengthened by the fact that in 1-MCP-treated fruits belonging to both class 0 and 1 there was a marked reduction of its mRNA (Fig. 2c).

*ACO2* expression was almost constitutive in the tested samples with a minimum in young (S1) fruit (Fig. 2a). Its steady state level was lower than that of ACO1 in all tested tissues, even in developing and maturing fruits, where ACO1 expression was at its minimum (see absolute quantification data of Fig. 5). Ethylene and, to a lesser extent, also NAA, slightly induced *ACO2* transcription in pre-climacteric S3II fruit (Fig. 2b). Surprisingly, a clear inductive effect of the 1-MCP treatment on ACO2 expression was observed in class 0 and, although to a lesser extent, also class 1 fruit (Fig. 2c). Besides the two known ones, three additional ACO genes were found in the peach genome and were named *ACO3* (ppa009228), *ACO4* (ppa022135m) and *ACO5* (ppa010361). *ACO4* is a truncated inactive and untranscribed version of *ACO1*, separated from it by less than 17 kilobases (kb). Among the peach *ACOs*, *ACO3* was the less expressed one in tested samples (see absolute quantifications in Fig. 5). It had a maximum in overripe fruit (i.e. S4II, Fig. 2a) and at S3II it was strongly induced by NAA (Fig. 2b). Given that its expression was very low and did not vary very much between control and treated samples, its responsiveness to 1-MCP, if any, was difficult to interpret (Fig. 2c). Expression of *ACO5* was highest at S2 and then decreases to be almost undetectable at ripening (Fig. 2a). Thus the slight variations observed after hormone treatments at S3II (Fig. 2b) and after 1-MCP application (Fig. 2c) were considered of limited physiological relevance.

### Transcriptional regulation of ethylene receptor genes

The developmental and hormonal (ethylene and NAA) control on the transcription of three known ethylene receptors was already known [4]. Here extensive search of the genome sequence allowed us to isolate only a fourth receptor, which was named ETR3 (ppa001846m, Additional file 4). As for the other receptor genes, also *ETR3* transcription raised with the progression of ripening to peak at S4 and decreased thereafter (Fig. 2a). As for *ETR1* and *ERS1*, neither ethylene nor NAA had a great impact on *ETR3* transcription, while ETR2 mRNA abundance increased after NAA and, mostly, ethylene treatment (Fig. 2b). 1-MCP had almost no effect on *ETR1*, it slightly down-regulated *ERS1* and *ETR3*, while it strongly suppressed *ETR2* transcription in both class 0 and class 1 fruit (Fig. 2c), thus confirming previous findings [25].

### Transcriptional regulation of genes belonging to the auxin domain

To further investigate the relationship between ethylene and auxin during peach fruit ripening, the expression of several genes belonging to the auxin domain was evaluated. Of the Aux/IAA genes shown to be up-regulated during peach ripening (Fig. 2a and [4]), five were induced by the ethylene inhibitor (CTG57, CTG84, CTG1741, CTG1727 and CTG671, see Fig. 2c). Interestingly, of these five genes, only three (i.e. CTG1741, CTG1727 and CTG671) were strongly induced by NAA at S3II (Fig. 2b), with the latter strongly up-regulated also by ethylene.

In addition, the transcription of two *TIR1* auxin receptors (i.e. CTG1541 and CTG2713) was abundant at ripening (Fig. 2a). Less clear was their ethylene and auxin responsiveness, as both genes were repressed by the hormones at S3II (Fig. 2b) and mildly regulated by 1-MCP (Fig. 2c). CTG1541 was induced while CTG2713 response depended on the class (repressed in class 0 and induced in class 1, Fig. 2c). A similar behavior was observed also for the ripening specific (Fig. 2a) and ethylene induced (Fig. 2b) *PIN1* (CTG3721) gene (Fig. 2c), thus confirming that class 0 and class 1 fruits behave differently [26].

Application of 1-MCP was almost ineffective on genes involved in auxin biosynthesis such as tryptophan synthase beta subunit (WS, CTG3371), and indole-3-glycerol phosphate synthase (IGPS, CTG3575), that were induced at ripening [4]. On the contrary, it was very effective in inducing the transcription of three previously uncharacterized genes (CTG134, CTG475 and CTG1993), two of which belong to the auxin domain.

Two genes whose products are involved in maintaining auxin homeostasis had a transcriptional profile almost overlapping with that of CTG134. In particular, CTG475 codes for an IAA amidohydrolase highly similar to Arabidopsis IAA-LEUCINE RESISTANT 1 (ILR1; [33]) and its abundance sharply increased during climacteric ripening (i.e. S4I and S4II, Fig. 2a). This gene was positively regulated by NAA and insensitive to ethylene (Fig. 2b); furthermore, it was stimulated by 1-MCP in both class 0 and 1 fruit (Fig. 2c). The second gene (CTG1993) codes for a GH3 protein, an IAA-amido synthase, and it was expressed almost exclusively during fruit ripening (Fig. 2a); its transcription was induced by NAA in pre-climacteric S3II fruit (Fig. 2b) and by 1-MCP, especially in class1 fruit (Fig. 2c).

### Transcriptional regulation of ripening-related transcription factors

Given the known importance of the role on ripening of transcription factors (TFs) belonging to different families, the expression of five genes, whose orthologs have been characterized in other systems [34], was tested. A SEPALLATA-like MADS-box (CTG1357), which is highly similar to tomato *RIN* [35], had the highest expression in S4II fruits (Fig. 2a), was induced by both ethylene and NAA at S3II (Fig. 2b), and seemed to be slightly repressed by 1-MCP in class 1 fruit (Fig. 2c). Similarly, a NAM TF (CTG1310), sharing strong similarity to tomato *NOR* [36], accumulated in mesocarp during ripening to peak at the end of the process (Fig. 2a), was induced by both ethylene and NAA at S3II (Fig. 2b), and seemed repressed by 1-MCP (Fig. 2c). Also two hormone-related TFs, the first mediating auxin (CTG1505, an ARF) and the second ethylene (CTG2116, an ERF) responses, had a ripening-related expression (Fig. 2a), but while the first was negatively regulated by both hormones at S3II, the latter was induced, especially by NAA (Fig. 2b). The unusual hormonal regulation of this ERF was confirmed by the 1-MCP treatment, which was ineffective on its expression, while the ARF responded differently in the two classes (Fig. 2c).

### Expression, structure, homology and putative function of CTG134

The gene (ppa012311m) corresponding to CTG134 was the only one to be highlighted by microarray analyses as induced at the S3II to S4I transition and by NAA and 1-MCP. This peculiar transcription profile was confirmed by qRT-PCR, which revealed that, besides in class 0, also in class 1 fruit 1-MCP induced its mRNA abundance (Fig. 2c). Moreover, the mRNA abundance of CTG134 was strongly increased by NAA and repressed by ethylene in pre-climacteric S3II fruit (Fig. 2b). In tissues other than ripening fruit at S4, CTG134 mRNA was hardly detectable (Fig. 2a).

**Fig. 3:**
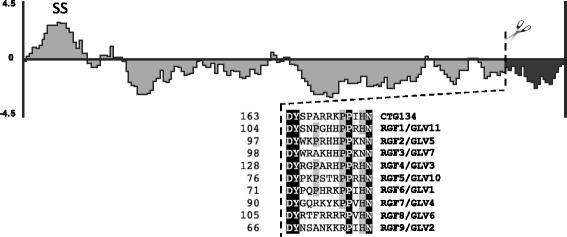
Structure of the CTG134 protein. Hydrophobicity plot of the protein sequence predicted from CTG134 (ppa012311m) and amino acid alignment of the C-ter with the corresponding part of some Arabidopsis RGFs/GLVs. The mature peptide hormone (*dark grey*) is released from the mature protein (*light grey*) after delivery in the cell wall (a signal sequence, SS, directs the protein to the secretion pathway)

The mRNA corresponding to CTG134 codes for a protein of 174 aa with a predicted molecular mass of 18.5 kDa. This polypeptide shares very low similarity with other plant proteins but for a small sequence of 13 amino acids (aa) at its carboxy terminus (C-ter). Like many other signaling peptides, this short hydrophilic protein has a predicted N-terminal sequence (Fig. 3) of about 23–24 aa that most likely directs it to the secretory pathway. The mature, apoplastic protein is rich in charged residues (32.9 %) and, although different in sequence, its structure resembles that of signaling peptides of the RGF/GLV type [37, 38]. The C-ter peptide sequence is highly conserved in a number of recently characterized Arabidopsis proteins (Fig. 3).

### 1-MCP increases free auxin levels in peach ripening fruits

**Fig. 4:**
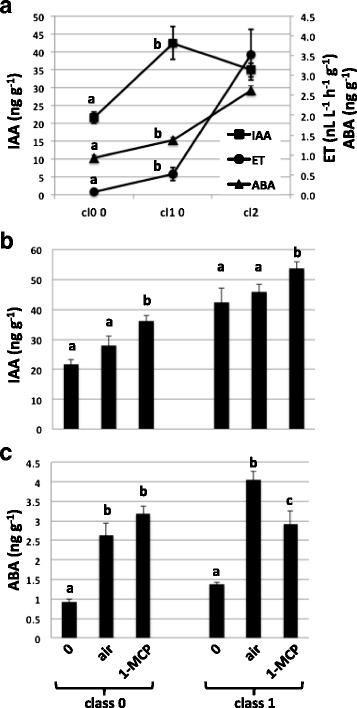
Auxin, ethylene (ET) and ABA levels during fruit ripening (panel (**a**)) and following 1-MCP treatment (IAA in panel (**b**), ABA in panel (**c**)). SRG peaches were sampled after 36 h of storage either in air or in 1-MCP (12 h) plus air (i.e. 24 h in air after the end of the 1-MCP treatment). Bars are the standard deviations from the means of three or more replicates. Letters above columns indicate significant differences with a Tuckey HSD test at *p* <0.05

As the transcription of several ripening- and IAA-induced genes was induced in 1-MCP-treated peaches, auxin was quantified in the same samples used for the RNA expression data of Fig. 2c and in class 2 fruit at harvest (time 0 of Fig. 1c; Fig. 4). The IAA concentration was lowest in class 0 fruit, reached a maximum in class 1 and slightly decreased thereafter (Fig. 4a). On the contrary, ethylene levels were hardly detectable in class 0 fruit, slightly increased in class 1 and peaked in class 2, thus showing that the auxin peak preceded that of ethylene (Fig. 4a). Also abscisic acid (ABA), long since known to accumulate in mesocarp of peach ripening fruits [39], and recently claimed to be among the determinants of ripening of several climacteric fruits [40, 41] including peach [42, 43], gradually increased from class 0 to class 2 fruit (Fig. 4a).

When the effect of 1-MCP on the IAA concentration was considered, it was clear that the ethylene inhibitor induced the amount of auxin in both class 0 and class 1 fruit (Fig. 4b). It has to be noted that, at the same time point (i.e. 24 h after the end of the treatment), 1-MCP did not alter ethylene production, but only its action (i.e. it delayed fruit softening, Fig. 1a).

Blocked ethylene perception did not significantly alter ABA concentration in class 0, while it reduced it in class 1 fruits (Fig. 4c).

### Timing and hierarchy of the hormonal signals during ripening

**Fig. 5:**
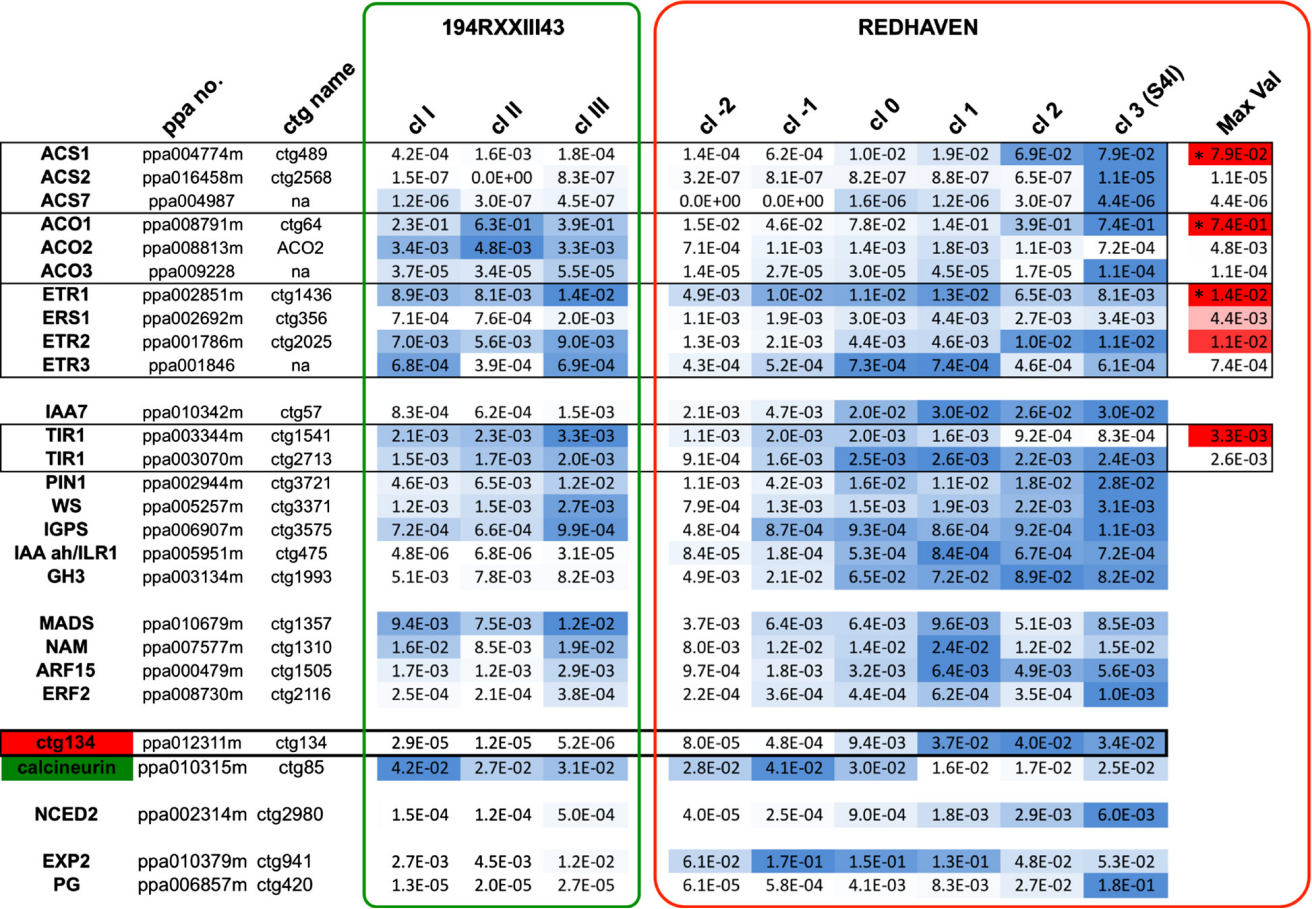
Absolute expression profiles of selected genes at the transition from maturation to ripening in melting flesh (Redhaven, RH) and stony-hard (194RXXIII43, RXX) genotypes. Gene groups and colors are as for Fig. 2 but for the last column (Max Val). As quantification was carried out with a standard, comparison of the relative abundance among members of the same gene family has been added (from white to red from the lowest to the highest, marked with an asterisk)

To better clarify timing and hierarchy of the hormonal cascade that leads to climacteric ripening (i.e. the switch form system-1 to system-2 ethylene synthesis), the S3II-S4I transition in melting flesh Redhaven (RH) peaches was investigated with a better temporal resolution than that of Fig. 2a, that spanned whole fruit development (i.e. 8 vs 120 days). Also in this case, fruits, collected on the same day, were correctly graded by means of their I_AD_ values into six classes (two for the harvest at 104 dAFB and four for that at 110 dAFB; see Additional file 5). Furthermore, fruits from a selection carrying the “stony hard” trait (194RXXIII43, RXX thereafter; Verde, personal communication), known for its inability to produce ethylene during ripening [44], were used and also grouped according to their I_DA_ values (Additional file 6). A subset of the genes used in Fig. 2 were selected as exemplificative of their groups (i.e. ethylene, auxin, TFs and cell walls, besides the hormone peptide CTG134 and the calcineurin CTG85, that are the two mRNAs with the unexpected transcription profiles evidenced by the microarray analysis) and the absolute quantification of their transcripts determined in the nine samples. In this experiment, the absolute mRNA abundance was determined to allow precise comparison between RXX and RH and, within the same genotype, among genes of the same families (Fig. 5). Of the genes involved in ethylene synthesis in RH, *ACS1* showed the strongest transcriptional repression in RXX fruits (Fig. 5). In RH, its ripening-induced expression started earlier than that of *ACO1*, whose expression, together with those of the other *ACOs*, was not significantly repressed in RXX fruit (Fig. 5). Among the genes of the IAA domain, it was the IRL1-like CTG475 mRNA that peaked in class 1 fruit, immediately before ACS1 rise. Also IAA perception was critical in class 0/1 fruit, as evidenced by the expression of TIR1/CTG2713, which, it has to be noted, was very similar to that of the ethylene receptors ETR1 and ETR3. However, while receptors and IAA biosynthesis genes were expressed at comparable amounts also in RXX fruit, this did not occur for *ILR1-like CTG475*, nor for *GH3* (CTG1993) and *IAA7* (CTG57), whose products are involved in IAA catabolism and signal transduction, respectively, and were induced by IAA. On the contrary, the expression of *ETR1*, *ETR2* and *ETR3* in RXX was similar, if not higher, to that found in RH. In addition, the expression of ripening related transcription factors showed the cruciality of class 1 (maximum expression of MADS CTG 1357, NAM CTG1310 and ARF15 CTG1505) stage, that we propose to be at the turning point of system-1 to system-2 ethylene synthesis. Moreover, the fact that the expression of the TFs is similar in the two genotypes supports that the stony hard trait is not due to alteration in their transcription, as it is for *CNR* in tomato [45]. Striking expression differences were found for *CTG134*, which was almost undetectable in RXX fruits. The NCED2 mRNAs gradually accumulated in RH fruits as ripening proceeded, while their levels in RXX were comparable to those found in class −1/1 in RH. Lastly, the cell wall genes confirmed many previous reports on their different transcriptional regulation, with *PG* expression strictly dependent on ethylene, while *EXP2* transcription, albeit peaking before the climacteric (Fig. 5) and being repressed by both ethylene and auxin [4], also needed a fruit in a ripening status that is incomplete in RXX.

### Different competences to auxin in preclimacteric fruit

**Fig. 6:**
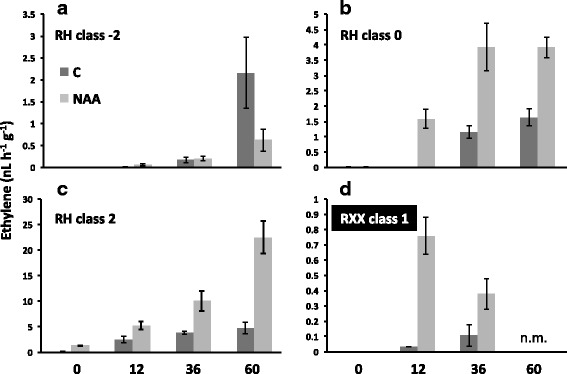
Effect of auxin treatment on the ability to produce ethylene in fruit at different ripening stages in melting flesh (Redhaven, RH, from class −2 to class 2, (**a**)–(**c**), respectively) and stony-hard (194RXXIII43, RXX, class 1, (**d**) genotypes. **c**: control, fruits treated with a mock solution; NAA: fruits treated with a solution containing NAA (1-naphthalene acetic acid, a synthetic auxin). Bars are the standard deviations from the means of three or more replicates

The effect of auxin on ethylene synthesis was tested on three classes of RH fruits (Fig. 6). On class −2 fruits (i.e. approximately comparable to S3II stage of Fig. 2), the synthetic auxin NAA had an inhibitory effect on ethylene synthesis (Fig. 6a). On the contrary, on class 0 and class 2 fruits auxin had a positive effect on ethylene production, being the induction stronger in class 0 after 12 h while the amplitude more pronounced on class 2 after 60 h from the treatment (Fig. 6b and c). Also class 2 fruits of the RXX genotype were able to produce ethylene after the NAA treatment, although the total amount of the hormone produced was much lower than that of the climacteric genotype (Fig. 6d), thus confirming recent findings [17].

**Fig. 7:**
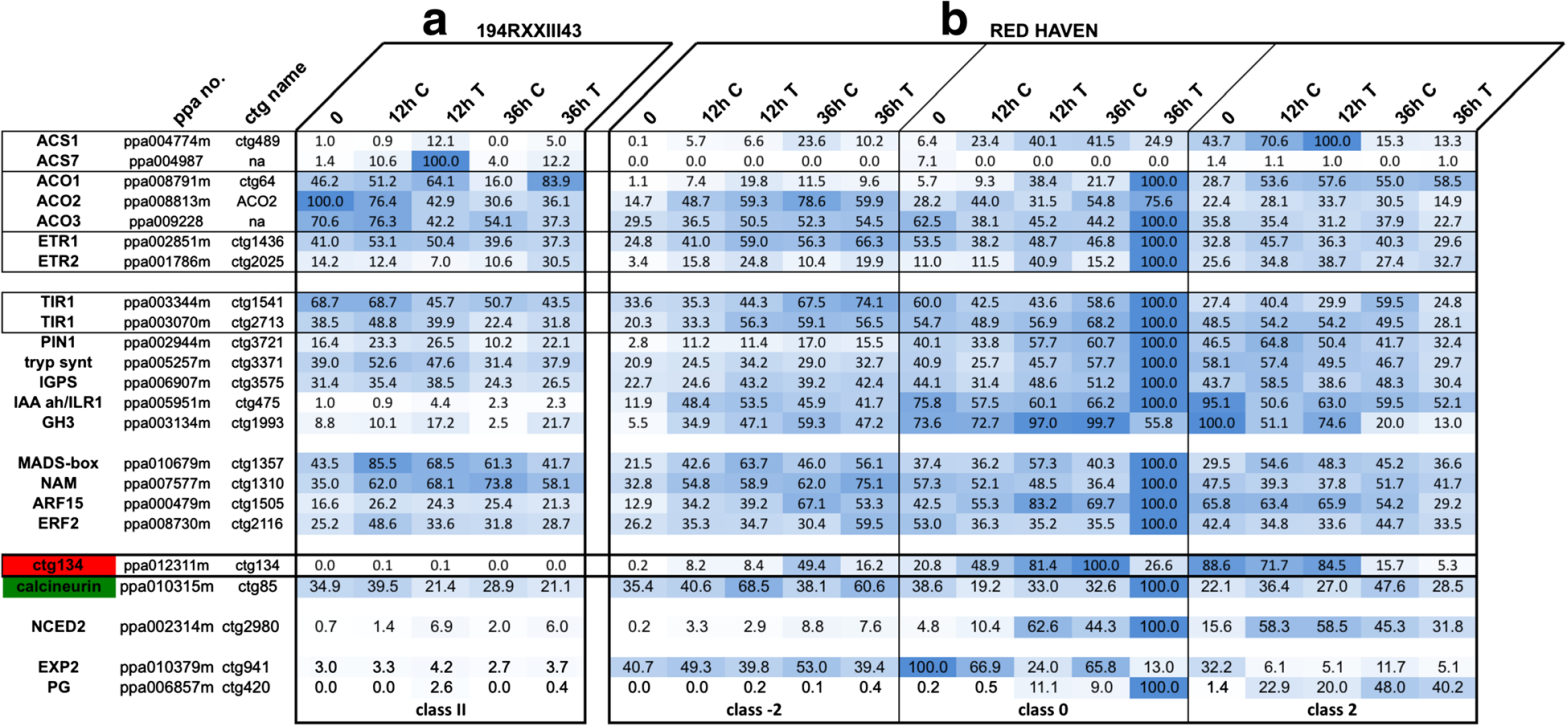
Relative expression profiles of selected genes following auxin treatment in fruit at different ripening stages in melting flesh (Redhaven, RH) and stony-hard (194RXXIII43, RXX) genotypes. Gene groups and colors are as for Fig. 2

The different behavior of class −2 compared to class 0 and class 2 fruit in RH was confirmed also at the transcriptional level (Fig. 7). Indeed, both *ACS1* and *CTG134* were repressed 36 h after the treatment in class −2, while they showed an opposite trend (induction at 12 h, repression at 36 h) in class 0 fruit (Fig. 7b). This opposite behavior was detected also in other auxin-inducible genes, as *GH3*, while ethylene regulated genes as *ACO1* and *ETR2* showed a marked up-regulation at both time-points in class 0 fruit (Fig. 7b), in agreement with the measured ethylene production (Fig. 6b). Ethylene biosynthetic genes *ACS1* and *ACO1* were more expressed in NAA treated RXX fruit (Fig. 7a). Also *ETR2* was more expressed 36 h after the treatment, while *ETR1* was not. The effectiveness of the NAA treatment was visible also on *GH3* and, albeit at a lower extent, also on *ILR1* gene expression, which were both induced, specially at 12 h, while expression of *CLB/CTG85*, which normally decreases during ripening, was higher in controls than in treated samples, meaning that the latter were riper. *NCED2* expression was induced by NAA both in RXX (Fig. 7a) and class 0 RH (Fig. 7b) fruits, but not in class −2. *PG* confirmed its strong dependency to ethylene for its expression, being repressed in class −2 and induced in class 0 RH fruits (Fig. 7b). The positive impact of NAA on ethylene synthesis in RXX fruits allowed a transient induction of *PG* expression (Fig. 7a). On the contrary, the pre-climacteric ethylene-independent expression of *EXP2* was confirmed in RH fruits (Fig. 7b). The only gene whose expression was almost undetectable in RXX fruits also after the NAA treatment was *CTG134* (Fig. 7a).

### DNA sequence of CTG134 in the RXX genotype

The expression of *CTG134* was absent in the stony hard genotype. For this reason, its sequence in this genotype was determined starting at 2782 bp before the ATG start codon down to 512 bp after the stop codon. The analyzed region did not contain any structural variation nor any polymorphism, thus being identical to the reference genome [30].

## Discussion

### Effect of 1-MCP on peach fruit ripening

The efficacy of 1-MCP in delaying peach fruit ripening is controversial. There are reports that both support an inhibitory action [20, 46] and others, which state that the chemical is (almost) ineffective [23–25]. Here we showed that its effects are largely dependent on the ripening stage at which the chemical is applied. We were able to make such a distinction due to the use of the non destructive Index of Absorbance Difference (I_DA_), which can estimate the fruit ripening stage by means of a computer-assisted spectrophotometric device [26]. Thus, if 1-MCP was supplied at an early ripening stage (in our case stage 0), a gross parameter such as pulp softening supports the chemical efficacy in delaying fruit ripening. On the other hand, the chemical was ineffective in delaying softening of class 2 fruits, thus indicating that the maturity stage of application is critical. Contrary to what happens in other fruit such as apple [47, 48], pear [49], tomato [50] but also in stone fruit as plum [51], in peach 1-MCP did not inhibit ethylene biosynthesis in class 1 and 2 fruits (Fig. 1b and c), thus confirming previous results [25, 46] but it did in class 0 fruits, where it was also effective in delaying softening (Fig. 1a). Thus, two apparently contradictory effects, which were seen at their best in class 1 fruits (Fig. 1b), were due to 1-MCP application on peaches: the delay of fruit softening, i.e. of the ethylene response, and the stimulation of ethylene production. Softening delay was efficient only when the ethylene evolution was low, probably because genes encoding cell wall degrading enzymes were induced with very low amount of hormone, as this has been shown for the tomato *PG* [52]. This finding might explain the contradictory reports on the effect of 1-MCP on both ethylene production and ripening delay in peach fruit present in the literature (reviewed in [18]).

Albeit not being useful as a post-harvest tool for the peach industry, the biological effect of 1-MCP was confirmed at the molecular level by transcriptome changes that the chemical could cause. Many (20 out of 63) 1-MCP inhibited genes were also ripening and ethylene induced, thus confirming previous findings [53, 54] about the importance of the hormone during peach ripening. On the contrary, 1-MCP had a positive effect on many ripening and ethylene repressed genes (18 out of 63), confirming its ability to delay the progression of the syndrome over a short time.

### Regulation of system-2 ethylene biosynthesis

System-2 ethylene production is largely dependent on the expression of *ACS1* [20, 29] and *ACO1* [32]. The expression of other members of the two families (*ACS2* and *ACS3* described in [29] and *ACO2* described in [32]) does not fit with the model of the transition from system-1 to system-2 proposed in tomato [13] and apple [55]. Of the four newly described putative ACS genes (see Additional file 3) only two (*ACS5* and *ACS7*) can be considered *bona fide* true ACSs, while *ACS4* and *ACS6*, being closely related to *AtACS10* and *AtACS12*, most likely lack ACS activity [31]. All ACS mRNAs but ACS1 were almost undetectable during fruit ripening. *ACS3* and *ACS5*, expressed in flower, could be involved in the ethylene production occurring during pollination [56] or organ shedding [57]. The unwanted wounding in the field might be the reason for the expression of the wound-inducible *ACS2* [29] in fully expanded leaves. *ACS5* and *ACS7* are expressed in fruits at early stages and thus it is possible that, together with *ACS2*, they are responsible for ethylene production in young fruits. Nonetheless, it is conceivable to exclude that they have a role similar to tomato *LeACS4* [58] or apple *MdACS3* [55] that, being expressed during the transition from system-1 to system-2, allow to rise ethylene concentration over the threshold necessary to start its autocatalytic production.

### Regulation of ethylene perception

During the initial phase of the transition from system-1 to system-2, a crucial role in sensing ethylene might be carried out by ETR1 and ERS1 whose expression increased at ripening but did not seem to be controlled by either ethylene or auxin, so that they could be considered under developmental control [4]. Only the expression of *ETR2* seemed to be associated with ripening in an ethylene-dependent manner. Indeed, when ethylene was not sensed, as in the presence of 1-MCP, *ETR2* transcription was strongly inhibited. In the peach genome sequence [30] only an additional ethylene receptor (i.e. ETR3) was isolated, thus bringing to only four the members of this family in peach, while there are five in Arabidopsis [59] and seven in tomato [34]. Nonetheless, ETR3 extremely low expression seemed to exclude a main role for it in ripening.

### Auxin homeostasis

The effect of auxin on ethylene production has not only been demonstrated in vivo but also in vitro. The ethylene amount produced by mesocarp disks cultured in vitro depends on both the concentration of NAA in the medium and the age of the fruits used to prepare the explants [60]. In particular, ethylene production was higher and faster if disks were obtained from fruits near ripening treated with 100 μM NAA. The increase of free auxin [17, 61] during the last stages of peach ripening might not rely only on *de-novo* synthesis but also on its release from conjugated forms. Indeed, the expression of CTG475 (Fig. 4b), coding for an IAA amidohydrolase of the ILR1 (IAA-LEUCINE RESISTANT 1) type, involved in the release of IAA from IAA-Leu (reviewed in [62]) correlates with free IAA content during ripening [17, 61] and following 1-MCP treatment (Fig. 3) better than that of genes involved in auxin biosynthesis (WS CTG3371 and IGPS CTG3575). However, we can not exclude that genes similar to Arabidopsis *TAA1* [63, 64] and *YUCCAs* [65, 66] also allow rapid and direct IAA synthesis from tryptophan also in peach and this will be investigated in the near future. To guarantee the correct auxin homeostasis, the expression of CTG475 seemed to be counterbalanced by that of CTG1993, coding for a GH3 protein. GH3 proteins have been demonstrated to be IAA-amido synthases which help to maintain auxin homeostasis by conjugating excess IAA to amino acids [67]. Both the genes encoding the IAA amidohydrolase (CTG475) and the GH3 (CTG1993) protein were strongly induced by NAA and expressed almost exclusively in S4 fruit. Furthermore, their expression was extremely low in the RXX genotype but it could be rescued by exogenous auxin (Fig. 5 and Fig. 7). This peculiar expression profile is very similar to that of *ACS1* and the expression of these three genes might be the cause of the peaks of both auxin and ethylene measured in S4 fruit [17, 61]. However, transcript profiling on more transition stages from pre-climacteric to climacteric fruits (Fig. 5) together with hormone quantifications (Fig. 4) allowed us to find that auxin peaked before ethylene production increased.

### 1-MCP induction of auxin-induced genes

The microarray experiment pointed out the peculiar regulation of CTG134 and CTG85, encoding a peptide hormone and a calcineurin B-like protein, respectively. The regulation of these genes was unexpected since most of those that are both ripening and auxin-induced are also 1-MCP-repressed while those that are ripening and auxin-repressed are also 1-MCP-induced. This is true for those genes whose auxin-regulation is indirect, because it is ethylene-mediated (i.e. auxin stimulates ethylene production that induces the expression of genes such as *ACO1*, *PG* and many others). However in peach there are also ripening-regulated genes directly responding to auxin [4]. Besides *CTG134*, lowering the stringency of the selection parameters pointed out that other genes belonging to the auxin domain and that were NAA-induced were 1-MCP-induced too.

The expression of some of these genes is often considered diagnostic for increased level of auxin in the tissue from which the RNA has been obtained. The expression profiles of genes such as those coding for GH3 (CTG1993) and Aux/IAA proteins (CTG1741, CTG1727, CTG671, CTG84 and CTG57) suggested that a rise in free auxin concentration had probably occurred in peaches following the 1-MCP treatment. Auxin measurements in fruits confirmed this hypothesis (Fig. 4) and, based on gene expression data, we propose that this increase is, at least partly, mediated by the activity of the amidohydrolase encoded by the *CTG475* gene.

The 1-MCP effects on ethylene synthesis depended on the physiological state of the peach fruits (inhibition in class 0, induction in class 1 and 2). In addition, the kinetic of the induction of transcription of genes belonging to the auxin domain were different in class 0 and class 1 fruit. Genes like *CTG134* and *CTG475* were strongly induced both in class 0 and in class 1 fruit, while others, such as *CTG1993* better responded in class 1 fruit. The different physiological status of fruits belonging to different I_DA_ classes were confirmed also in RXX and RH peaches. It was clarified that auxin has an inductive effect on ripening only if a given maturation state is reached, otherwise it inhibits the process. The fact that blocking ethylene perception stimulated the auxin synthesis needed for the system-2 ethylene production tells about the importance of an intact receptor apparatus also in peach, as it has been shown by inverse genetics in tomato [68]. These differences could be appreciated only because the fruit sorting was finely tuned due to the use of the I_AD_ index [26] and probably are the results of a cascade of signals leading to fruit ripening.

### Interactions with other hormones

The increase of ABA content during peach ripening has long since been known and considered to be dependent on ethylene [39]. The idea of a possible ethylene control on ABA synthesis is here strengthened by the fact that *NCED2* (CTG2980), but also *CTG75* (Additional file 2), was strongly inhibited by 1-MCP, besides being induced by ripening, ethylene and NAA. However it has also been reported that ABA reach its height before the ethylene peak [42] and recently it has been shown that, if applied when competence to ripening has been acquired, ABA has an inductive effect, besides on ripening, also on the regulation of ethylene biosynthetic and auxin responsive genes [43]. This extended hormone cross-talk could have even a wider relevance since it has been observed also in roots of cleavers. In the latter system *NCED* expression was directly induced by IAA and sustained by ethylene, so that both contributed to an induced ABA synthesis [69]. In peach it could be that ethylene and ABA control each other to synchronize different aspects of ripening.

### A model for the transition of ethylene biosynthesis from system-1 to system-2 in peach

There are several scenarios that might be hypothesized in which the actors and their interaction that lead to the transition from system-1 to system-2 ethylene production might be placed. Based on expression profiles of ripening related genes and their responsiveness to ethylene, 1-MCP and NAA in melting flesh (RH and SRG) and stony hard (RXX) genotypes, we propose that the transition is initiated, together with other not yet characterized developmental signals such as the activity of TFs [28], by the increase in free auxin levels, possibly at a stage similar to class 1 in RH (Fig. 5). *ACS1* transcription, that might depend also on RIN-like MADS, as observed in tomato [70], is the rate limiting step of system-2 ethylene synthesis, and can rely only on auxin increase but not on other *ACS* genes expression to switch ethylene synthesis from system-1 to system-2. Since ethylene production must be controlled, a possible feed-back regulation involves the hormone sensing carried out by ETR1, ERS1, ETR2 and possibly ETR3. The newly synthesized ethylene negatively regulates the mRNA abundance of amidohydrolase CTG475 but not of GH3 CTG1993, thus allowing a balanced auxin homeostasis. When ethylene concentration is low (i.e. class 0 SRG or class −2 to class 1 RH fruits) or receptors are blocked by 1-MCP, fruit cells that have completed their maturation sense that ethylene is missing, so its synthesis has to be induced by a release of free auxin. The *CTG134* gene has several features that make it a good candidate as a possible component of the rheostat that balances auxin and ethylene synthesis. Its NAA induced expression is almost exclusively limited to fruit at early S4 and inhibited by ethylene. And when ethylene should be there (i.e. in S4 fruit) but it is not sensed because of the presence of 1-MCP, *CTG134* transcription raises very quickly, especially in fruit at the stage in which there is the transition from system-1 to system-2 (class 0 in the SRG experiment, class 1 in the RH one). The regulation of the expression of the peptide encoded by *POLARIS* (*PLS*) in Arabidopsis (i.e. induced by auxin and repressed by ethylene) and its involvement in a regulatory loop of auxin–ethylene interactions indicates that the cross-talk between ethylene and auxin can be mediated by signaling peptide [71]. The 13 aa sequence at the C-terminus of the CTG134 protein is highly conserved with Arabidopsis RGF/GLV like peptides [37, 38]; thus, besides being involved in root meristem maintenance [37] and in root gravitropic response [38] RGF/GLV peptides are involved also in fruit ripening. The ability of those peptides to control auxin distribution and to reinforce its action by regulating the turnover of an auxin efflux carrier [38], thus regulating auxin gradients, might explain the ethylene induced expression of CTG3721, coding for a peach PIN1. As ripening usually commences in several regions of a fruit, the predicted apoplastic localization of CTG134, together with ethylene diffusion, might help the spreading of ripening from cell to cell throughout the fruit.

Altogether these data lead to the hypothesis that the transition from system-1 to system-2 ethylene biosynthesis in peach fruit is controlled by a regulatory loop of auxin-ethylene interactions in which hormone levels are reciprocally controlled by a signaling system involving a RGF/GLV peptide hormone.

## Conclusions

Here we showed that blocking ethylene receptors with 1-MCP increases free auxin content in ripening peach fruit, thus leading to ethylene overproduction. This increase is sustained by the transcriptional activation of *ILR1-like CTG475* and thus, at least partly, by auxin de-conjugation. The CTG134 protein, a precursor of a peptide hormone of the RGF/GLV type is a good candidate to mediate this ethylene-auxin cross-talk. The auxin-dependent rise in ethylene concentration represses many of the auxin genes, among which also *ILR1-like CTG475*, and that coding for *CTG134*. This new player opens the theoretical possibility to design new rational and environmentally friendly agrochemicals useful to control ripening in those crops, as peach, where 1-MCP is ineffective and cold storage has many drawbacks.

## Methods

### Plant material

Fruits were coming from four collections from three different fruit cultivars/genotype, carried out on different seasons. Fruit of the melting flesh type were from *Prunus persica* L. Batsch, cv. ‘Redhaven’, RH, and from cv. ‘Stark Red Gold’, SRG. SRG is a nectarine, but, being the nectarine phenotype dependent on a mutation on a single gene [72], for sake of convenience and to avoid confusion to readers not acquainted with the *P. persica* system, also SRG fruits are here called peaches. The third genotype was a selection, called 194RXXIII43 (RXX thereafter), carrying the “stony hard” trait (Verde, personal communication), known for its inability to produce system-2 ethylene during ripening [44], because of its incapacity to accumulate high levels of auxin [17].

RH peaches were collected from 7-year-old trees, grafted on seedling rootstock and trained to an open-vase shape, grown at the experimental farm of the University of Padua, Italy. SRG and RXX peaches were harvested from 8-year-old trees, grafted on seedling rootstock and trained to a Y shape, grown at the experimental farm of the University of Bologna, Italy. For each cultivar, the double sigmoid growth pattern was established based on fruit diameter, which was monitored weekly on 40 fruit during the growth cycle. The first derivative was calculated in order to discriminate the four growth stages S1-S4 [73, 74] (Additional file 5). RH peaches at different stages of development [first collection, i.e. S1, S2, S3I, S3II, S4I, and S4II, corresponding to 40, 65, 85, 95, 115 and 120 days after full bloom (dAFB), respectively] were collected and treated or not (controls) with auxin or ethylene (see below) and used in experiments presented in Fig. 2a and b. From the same trees, fully expanded leaves, without any evident signs of senescence, and flowers at full bloom were collected, frozen in liquid nitrogen and stored at −80 °C for subsequent use. SRG peaches (second collection, used in microarray experiments and in those presented in Figs. 1, 2c and 4) were harvested at 123 dAFB (S4), i.e. at commercial maturity date, which is about two weeks later than that of RH. In order to obtain homogeneous fruit at different stages of ripening, fruits were graded immediately after harvest into 3 classes by decreasing ranges of the index of absorbance difference (I_AD_; class 0: I_AD_ 1.2-0.9; class 1: I_AD_ 0.9-0.6; class 2: I_AD_ 0.6-0.3), as previously described [26]. The I_AD_ is a non-destructive marker of peach fruit ageing which is calculated as the difference in absorbance between two wavelengths near the chlorophyll-*a* absorption peak (670 and 720 nm; [26]). According to previous studies [26], fruit from the 3 classes could be classified as belonging to pre-climacteric (class 0), onset of climacteric (class 1), and full climacteric (class 0) stages of the ripening process. Fruits from each class were treated or not (controls) with 1-methylcyclopropene (1-MCP) as described below. To zoom into the ripening process and take advantage of the I_AD_, two additional samplings (third collection, used in experiments presented in Figs. 5, 6 and 7) of RH fruit were carried out at 104 and 110 dAFB. After I_AD_ grading, fruit collected at 104 dAFB (roughly corresponding to S3II of the first collection) were assigned to classes −2 and −1, while fruit collected at 110 dAFB were divided into four classes, from 0 to 3 (see Additional file 5 for the sampling scheme of RH fruit). To take advantage of the well-characterized “stony hard” model, a fourth collection was conducted at 105 dAFB and peaches were sorted according to their I_AD_ values (see Additional file 6). Worth to mention that the I_AD_ value is a continuous parameter and thus class assignment depended on the number of the classes. For the second and fourth collection S4 fruit was split into three classes, while for the third into four classes.

### Hormone treatments on Redhaven and 194RXXIII43 fruit

The ethylene treatment was provided by placing whole fruit (attached to a branch) in a sealed chamber and flushing them with ethylene (10 μL L^−1^) in air at a flow rate of approximately 6 L h^−1^. The auxin treatment was performed by dipping the whole fruit in 1-naphthalene acetic acid [NAA, 2 mmol L^−1^ added with Silwet L-77 (200 μL L^−1^) as surfactant] for 15 min; thereafter, fruit were sprayed with the NAA solution every 12 h over a period of 48 h (NAA omitted in the mock control).

### 1-MCP treatments on Stark Red Gold fruit

One hundred fruit per class were placed in two sealed 30-L plastic jars (50 fruit each). SmartFresh™ (AgroFresh Inc., Philadelphia, PA, USA), a commercial powder containing 0.14 % (w/w) 1-MCP a.i., was prepared as a 10-fold concentrated stock solution following the technical bulletin of the company, and injected as 10 mL of air (final concentration 1 mL L^−1^ equivalent to 1 μL L^−1^). On the same experimental conditions, 100 fruit belonging to classes 1 and 2 were incubated also with 5 μL L^−1^ 1-MCP. The same total number of fruit per class was kept in two sealed jars for 12 h at 25 °C without 1-MCP (air controls). At the end of treatments, temperature, ethylene and CO_2_ concentration within the jars were determined. Treated and control fruit were then transferred to a growth chamber at 25 °C. At the end of treatment (12 h) and at each following sampling time, ethylene production and flesh firmness were assessed on 20 control and 20 treated fruit. For molecular analyses, mesocarp tissues from a pool of 10 fruit per class were frozen in liquid nitrogen and stored at −80 °C until used.

### Ethylene production and flesh firmness determination

Ethylene production was measured by placing each fruit in a 1 L jar sealed with an air-tight lid equipped with a rubber stopper, and left at room temperature for 1 h. A 10 mL gas sample was taken and injected into a Dani HT 86.01 (Dani, Milan, Italy) packed-gas chromatograph as previously described [73].

Flesh Firmness (FF) was measured on the two opposite sides of each fruit, after removing a thin layer of the epicarp, using a pressure tester (EFFE.GI, Ravenna, Italy) fitted with an 8 mm diameter plunger.

Data on ethylene production and FF are given as the mean (*n* = 40) ± standard deviation of the population (SD). They were analyzed by Student’s *t*-test or one-way ANOVA procedures using the SAS Statistical Software (SAS Institute, Cary, NC, USA); means were separated by using the Newman-Keuls multiple range test at 5 % level.

### Extraction and purification of IAA and ABA

The extraction and purification of IAA and ABA was performed as previously described [75] with some modifications. For each biological replicate at least three frozen peaches were homogenized in liquid nitrogen and 0.5 g were then resuspended in cold (4 °C) 80 % methanol containing 100 μg butylated hydroxy-toluene and the internal standards D5-IAA (60 ng, Sigma) and D6-ABA (300 ng, Sigma). The mixture was stirred overnight at −20 °C. After centrifugation at 5000 *g* for 30 min at 4 °C, the extract was adjusted to pH 8.0 with ammonia and reduced to the aqueous phase under vacuum using a rotary evaporator (rising film evaporator, RFE) with a water bath temperature of 35 °C. The aqueous phase was centrifuged (5000 *g* for 30 min at 4 °C), the supernatant was adjusted to pH 2.5–3.0 with 2 M acetic acid and then extracted three times in 5 mL of ethyl acetate. The organic layers were combined and evaporated to dryness under vacuum using a RFE with a water bath temperature of 35 °C. Extracts were dissolved in 5 mL 0.1 M acetic, set aside for 1 h and passed through a C18 Waters Sep Pak cartridge that had been pre-equilibrated with 5 mL of a solution 50 % (v/v) methanol and 50 % (v/v) 0.1 M acetic acid. After washing the columns with 5 mL 17 % methanol, elution of the two phytoregulators were performed through 5 mL 40 % (v/v) methanol and 60 % (v/v) 0.1 M acetic acid. The eluates were adjusted to pH 8 with ammonia and then evaporated to dryness (40 °C) overnight. The dried eluates were derivatized for 6 h with 150 μL di N-Methyl-N-(trimethylsilyl) trifluoroacetamide (Sigma) at 30 °C and analyzed through GC-MS. For each sample 3 biological replicates and 2 technical ones were obtained.

### Quantification of IAA and ABA

GC-MS analyses were conducted using a 7890/5975-MSD GC-MS (Agilent Technologies) injecting 2 μL of the derivatized samples in splitless mode with a CTC-PAL auto sampler. The column (DB-5, 30 m x 0.25 mm, 0.25 μm, Agilent Technologies), under a constant flow of 1 ml min^−1^ using high purity helium as carrier gas, was heated 1 min at 70 °C, 6 min ramp to 76 °C, 45 min ramp to 350 °C, 1 min at 350 °C, 10 min at 330 °C. The ionization was for electron impact at −70 eV and the temperatures of MS Source and Quad were held at 230 and 150 °C, respectively.

The acquisition was carried out in Selected Ion Monitoring mode, following, with a dwell time of 20 msec, the ions with m/z 324 (RT = 30.75 min) and 202 (RT = 30.80 min) for D5IAA and IAA and with m/z 194 (RT = 34.28 min) and 190 (RT = 34.35 min) for D6ABA and ABA, respectively. For IAA quantification the relationship emerging from a curve of seven different concentration ratios of standard samples of IAA/D5IAA was employed.

Spectral integration was performed using the software MET- IDEA v. 2.08 [76] while for the statistical Tuckey HSD and t-Student tests the software STATISTICA 8.0 (StatSoft Inc.) was employed.

### RNA extraction

Each sample was prepared from a frozen powder obtained by grinding mesocarp sectors from at least four different fruits. From four grams of this powder, total RNA was extracted following a protocol previously described [77]. RNA yield and purity were checked by means of UV absorption spectra, whereas RNA integrity was ascertained by electrophoresis in agarose gel followed by ethidium bromide staining.

### Microarray experiments

Microarray experiments were carried out by retrotranscribing 15 μg of total RNA. The obtained cDNAs were labelled with Cy3 and Cy5 dyes (GE Healthcare, USA) and competitively hybridized to oligonucleotide microarrays platform μPEACH1 (GEO ID: GPL8584). Microarrays were read with the ScanArray LITE confocal laser scanner (PerkinElmer, USA) and the values extracted with Spotfinder 3.1 [78] as previously described [4].

Normalized data were loaded in MeV 3.1 [78] and subjected to SAM (Significance Analysis of Microarrays, [79] analyses. Since the comparison (Class 0 36 h air → Class 0 36 h 1-MCP,) was repeated twice and two “swap” experiments have been carried out too, there were 4 values for each gene to be used in the SAM analysis. Lists of clones with significant changes in expression were identified at delta values that gave a false discovery rate (FDR) of 5 %.

The data discussed in this publication have been deposited in NCBI's Gene Expression Omnibus and are accessible through GEO Series accession number GSE16224 [80].

### Expression analyses by quantitative Real time PCR (qRT-PCR)

qRT-PCR was performed and the obtained data manipulated as previously described [4]. Briefly, 6 μg of total RNA for each sample, pre-treated with 1.5 units of DNaseI, was converted to cDNAs by means of the “High Capacity cDNA Archive Kit” (Applied Biosystems), which uses random hexamers as primers. Primer sequences for the selected genes are listed in Additional file 7. Oligonucleotides DZ79: TGACCTGGGGTCGCGTTGAA and DZ81: TGAATTGCAGAATCCCGTGA annealing to the Internal Transcribed Spacer of the ribosomal RNA, have been used to amplify the reference gene. Reactions were carried out using 25 μL of the “Syber green PCR master mix” (Applied Biosystems), with 0.05 pmoles of each primer, in the “7500” instrument (Applied Biosystems). The obtained threshold cycle (CT) values were analysed by means of the “Q-gene” software [81] by averaging three independently calculated normalized expression values for each sample. Expression values were given as mean of the normalized expression values of the triplicates, calculated according to equation 2 of the “Q-gene” software [81]. Differences in expression values among probes reflect different quantities of target amounts. For some genes, slightly different expression values were registered in fruit at similar ripening stages (e.g. S4I in Redhaven and Class 0 in Stark Red Gold). These light discrepancies were probably due to year-dependent natural fluctuations or to the different genotypes of nectarine and peach fruit.

Numerical values obtained with these calculations were transformed into graphics by means of the “GraphPad” software (GraphPad Software, USA).

### Availability of supporting data

The microarray data supporting the results of this article are available in the NCBI's Gene Expression Omnibus repository, and are accessible through GEO Series accession number GSE16224 [80].

## Additional files

Additional file 1:
**Excel file with the microarrary data.** (XLS 21 kb) Additional file 2:
**Figure with qRT-PCR validation of microarray data.** (PDF 102 kb) Additional file 3:
**Figure with the phylogenetic analysis of ACS proteins.** (PDF 183 kb) Additional file 4:
**Figure with the phylogenetic analysis of ethylene receptor proteins.** (PDF 257 kb) Additional file 5:
**Figure with the sampling and grading of RH fruit used in Figs.** **2** **,** **3** **,** **6** **and** **7** **.** (PDF 1207 kb) Additional file 6:
**Table with the sampling and grading of 194RXXIII43 fruit used in Figs.** **3** **,** **6** **and** **7** **. **(PDF 36 kb) Additional file 7:
**Table with the list of oligonucleotides used in the qRT-PCR experiments.** (XLS 21 kb)
