# Supplementary material for: On the role of ethylene, auxin and a GOLVEN-like peptide hormone in the regulation of peach ripening
Source: BMC Plant Biol. 2016 Feb 11;16:44. doi: 10.1186/s12870-016-0730-7 (PMC4750175; doi:10.1186/s12870-016-0730-7)
Supplement: Additional file 2: — Figure with qRT-PCR validation of microarray data. (PDF 102 kb) [file 12870_2016_730_MOESM2_ESM.pdf]

Additional file 2

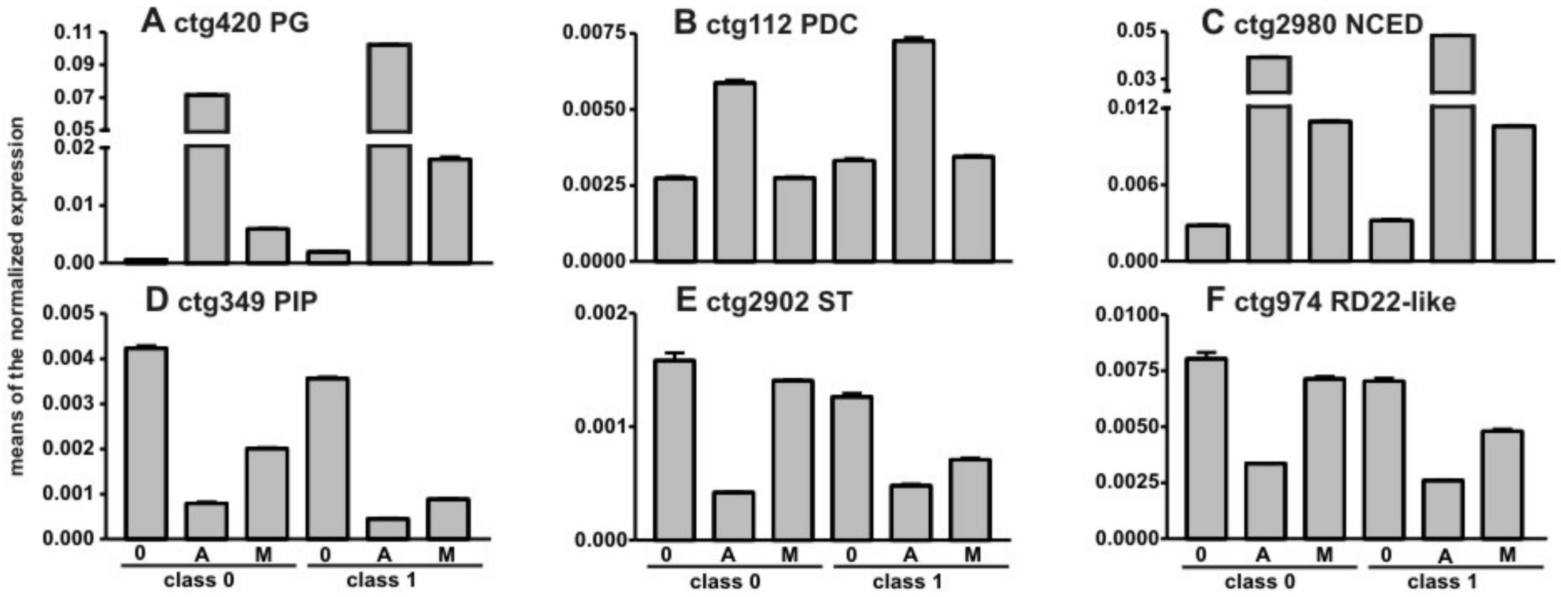

Additional file 2. Relative expression profiles of some genes either induced by ripening, ethylene and auxin and repressed by 1-MCP (PG: polygalacturonase, ctg420; PCD: pyruvate decarboxylase, ctg112; NCED: nine-cis-epoxycarotenoid dioxygenase, ctg2980) or repressed by ripening ethylene and auxin and induced by 1-MCP (PIP: plasma membrane intrinsic protein, ctg349; ST: sorbitol transporter, ctg2902; RD22: dehydration-induced protein RD22-like, ctg974) analysed in Class 0 and Class 1 nectarines following 1-MCP treatments. Values have been obtained by means of qRT-real-time and are given as mean of the normalized expression values of the triplicates, calculated according to equation 2 of the "Q-gene" software [80]. 0: time 0, start of the 1-MCP treatment; A: air controls; M: 1-MCP treated fruit.
