# Supplementary material for: On the role of ethylene, auxin and a GOLVEN-like peptide hormone in the regulation of peach ripening
Source: BMC Plant Biol. 2016 Feb 11;16:44. doi: 10.1186/s12870-016-0730-7 (PMC4750175; doi:10.1186/s12870-016-0730-7)
Supplement: Additional file 4: — Figure with the phylogenetic analysis of ethylene receptor proteins. (PDF 257 kb) [file 12870_2016_730_MOESM4_ESM.pdf]

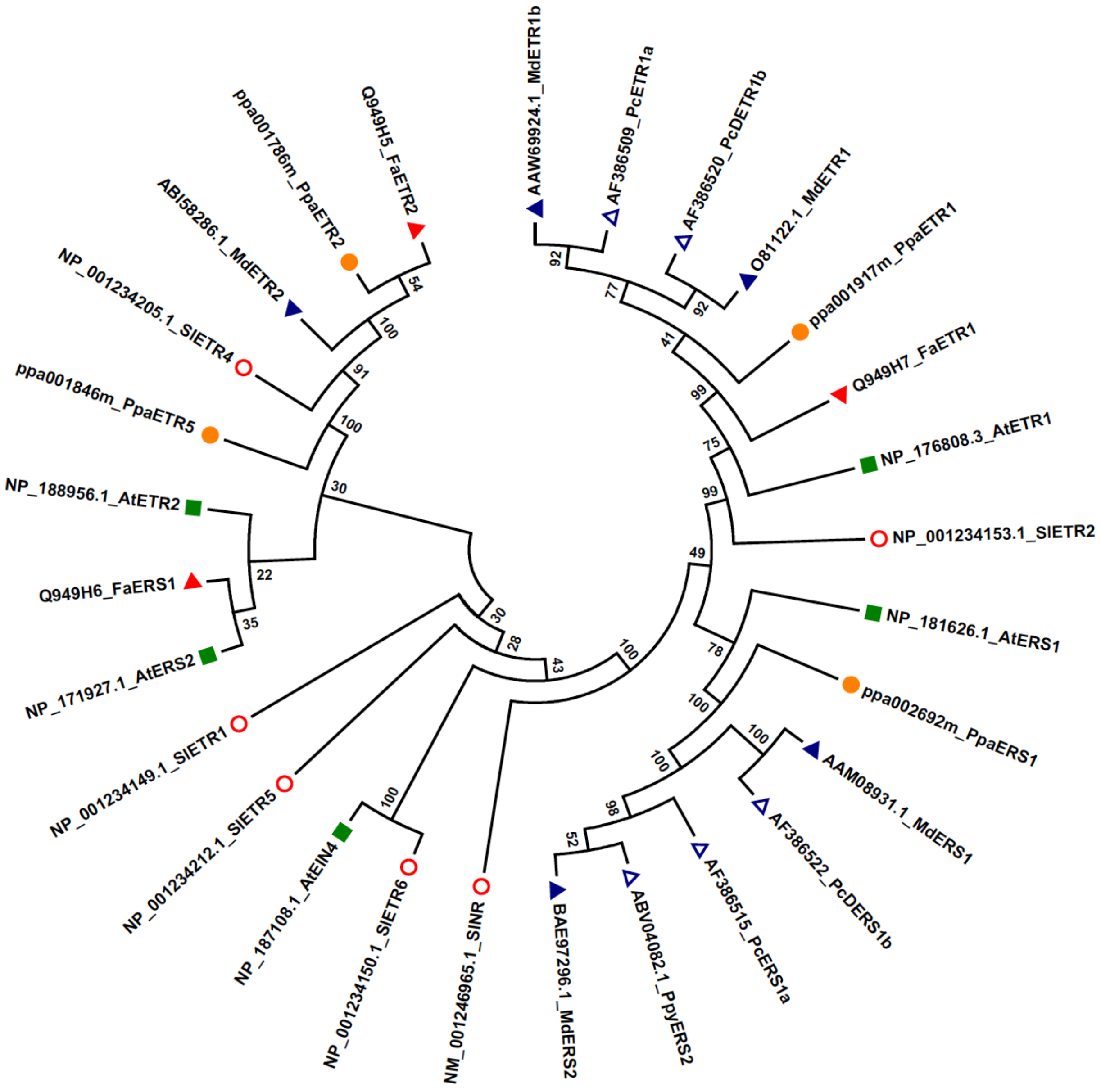

Additional file 4: ethylene receptors' phylogenetic tree

Additional file 4. Phylogenetic analysis of ethylene receptor proteins. Phylogenetic and molecular evolutionary analyses were conducted using MEGA version 5 [1]. The evolutionary history was inferred using the Neighbor-Joining method and 1000 bootstrap replicates (values at nodes) [2]. The optimal tree with the sum of branch length is shown. The tree is drawn to scale, with branch lengths in the same units as those of the evolutionary distances used to infer the phylogenetic tree. The evolutionary distances were computed using the Poisson correction method [3] and are in the units of the number of amino acid substitutions per site. The analysis involved 28 amino acid sequences. All positions containing gaps and missing data were eliminated. There were a total of 340 positions in the final dataset. Same symbol used for sequences belonging to the same species.

1. . Tamura K., Peterson D., Peterson N., Stecher G., Nei M., and Kumar S. (2011). MEGA5: Molecular Evolutionary Genetics Analysis using Maximum Likelihood, Evolutionary Distance, and Maximum Parsimony Methods. Molecular Biology and Evolution 28: 2731-2739.

2. Saitou N. and Nei M. (1987). The neighbor-joining method: A new method for reconstructing phylogenetic trees. Molecular Biology and Evolution 4:406-425.

3. Zuckerkandl E. and Pauling L. (1965). Evolutionary divergence and convergence in proteins. Edited in Evolving Genes and Proteins by V. Bryson and H.J. Vogel, pp. 97-166. Academic Press, New York.
