# Supplementary material for: On the role of ethylene, auxin and a GOLVEN-like peptide hormone in the regulation of peach ripening
Source: BMC Plant Biol. 2016 Feb 11;16:44. doi: 10.1186/s12870-016-0730-7 (PMC4750175; doi:10.1186/s12870-016-0730-7)
Supplement: Additional file 5: — Figure with the sampling and grading of RH fruit used in Figs. 2 , 3 , 6 and 7 . (PDF 1207 kb) [file 12870_2016_730_MOESM5_ESM.pdf]

Additional file 5. Sampling and grading of peach fruit in developing (A) and ripening SRG (B) and RH (C) classes.

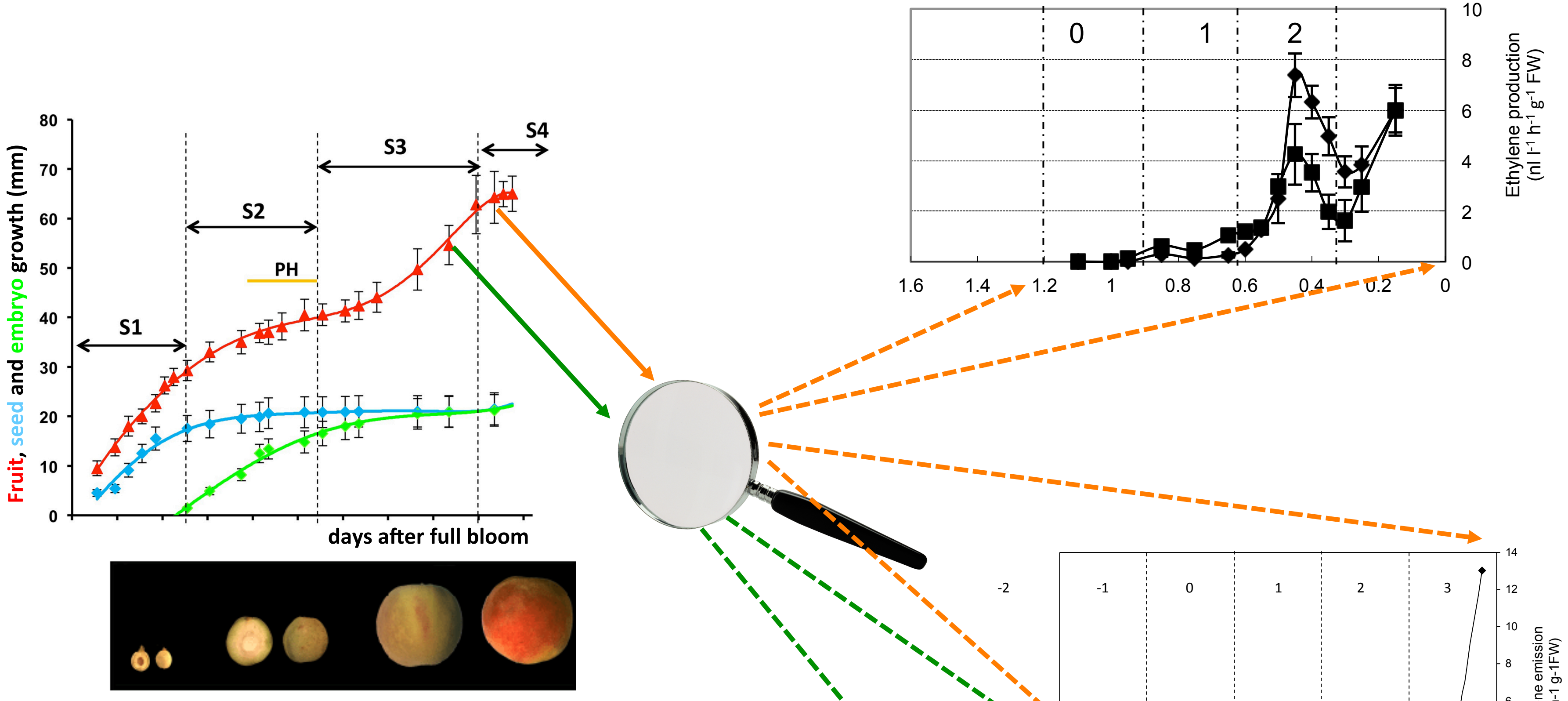

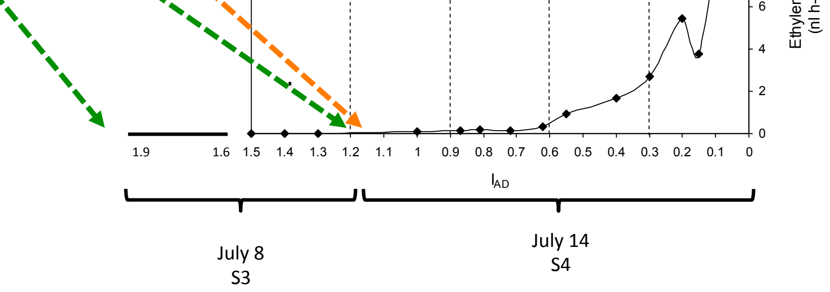

Additional file 5. Sampling and grading of peach fruit in developing (A) and ripening SRG (B) and RH (C) classes.

A: Fruit growth (red) is expressed as cross diameter while length is used for seed (blue) and embryo (green) development. Fruit developmental cycle has been divided into four main stages (S1 to S4) according to the first derivative of the fruit growth curve. The yellow horizontal line indicates pit hardening (PH). Modified from 1. Bonghi C, Trainotti L, Botton A, Tadiello A, Rasori A, Ziliotto F, Zaffalon V, Casadoro G, Ramina A: **A microarray approach to identify genes involved in seed-pericarp cross-talk and development in peach**. *BMC Plant Biology* 2011, **11**:107.

B: staging of SRG fruits collected at S4 according to I_{AD} .

B: staging of RH fruits collected at S3 (green arrows) and S4 (orange arrows) according to I_{AD} . Intervals are defined as 0.3 I_{AD} units and related to ethylene evolution.
