# Supplementary material for: On the role of ethylene, auxin and a GOLVEN-like peptide hormone in the regulation of peach ripening
Source: BMC Plant Biol. 2016 Feb 11;16:44. doi: 10.1186/s12870-016-0730-7 (PMC4750175; doi:10.1186/s12870-016-0730-7)
Supplement: Additional file 6: — Table with the sampling and grading of 194RXXIII43 fruit used in Figs. 3 , 6 and 7 . (PDF 36 kb) [file 12870_2016_730_MOESM6_ESM.pdf]

**Class**	**I_{AD}**	**FF (N)**	**SSC (°Brix)**	**Ethylene (nL• h^{-1}• g^{-1}FW)**
**1**	0.6-0.4	53 ± 2	11.2 ± 0.30	n.d.
**2**	0.4-0.2	50 ± 2	11.1 ± 0.40	n.d.
**3**	0.2-0	51 ± 2	12.6 ± 0.1	traces in some fruits

Supplementary Table 2

Table S2. Sampling and grading of 194RXXIII43 fruit in ripening classes according to I_{AD} . FF: Flesh firmness, in Newtons. SSS: Soluble Solid Content, expressed as °Brix
